# Impact of Mango Bagasse and Peel Confectionery Rich in Dietary Fiber on Gut Microbiota, Metabolite Profiles, and Genetic Regulation in High-Fat-Diet-Fed Wistar Rats

**DOI:** 10.3390/nu17233780

**Published:** 2025-12-02

**Authors:** Yuritzi Barbosa, Marcela Gaytán-Martínez, Rocio Alejandra Chavez-Santoscoy, Erika Magallón-Gayón, Silvia Hinojosa-Alvarez, Adriana Chico-Peralta, Marcos de Donato, Aurea K. Ramírez-Jiménez

**Affiliations:** 1 Tecnologico de Monterrey, School of Engineering and Science, Av. Eugenio Garza Sada 2501 Sur, Monterrey 64700, NL, Mexico; 2 Programa de Posgrado en Alimentos del Centro de la República (PROPAC), Research and Graduate Studies in Food Science, School of Chemistry, Universidad Autónoma de Querétaro, Centro Universitario, Cerro de las Campanas S/N, Santiago de Queretaro 76010, QRO, Mexico

**Keywords:** PPAR pathway signaling, fiber intake, mango by-products, gut microbiota, transcriptomics

## Abstract

Background/Objectives: Insufficient dietary fiber intake contributes to gut microbiota dysbiosis, systemic inflammation, and the onset of obesity-related metabolic disorders. Agro-industrial by-products have emerged as sustainable sources to restore microbial and metabolic balance. This study aimed to evaluate the effects of a mango bagasse- and peel-based confectionery (MC) on gut microbiota composition, short-chain fatty acids (SCFAs), and hepatic gene expression in Wistar rats fed either a standard diet (STD) or a high-fat diet (HFD). Methods: Twenty-four rats were randomly assigned to four groups (STD, MC-STD, HFD, MC-HFD) and treated for 11 weeks. Eating behavior, body composition, microbiota composition, SCFAs, and hepatic transcriptomics were evaluated. Results: MC supplementation did not significantly alter weight gain or SCFA levels but shifted clustering patterns in principal component analysis, indicating a distinct dietary response. Microbiota analysis revealed a trend toward lower relative abundances of obesogenic species such as *Phascolarctobacterium faecium* and *Ruminococcus torques*, while *Intestimonas butyriciproducens* and *Anaerostipes hadrus* were modulated according to diet type. Transcriptomic profiling demonstrated consistent downregulation of lipid metabolism–related genes (*Cyp4a14*, *Hmgcs1*, *Cyp51*, *Fads1*), linked to PPAR signaling pathways. Conclusions: MC supplementation may beneficially modulate the gut–liver axis and highlights the nutritional potential of fruit by-products as functional ingredients to promote metabolic health under high-fat dietary conditions.

## 1. Introduction

A persistent deficiency in dietary fiber intake has been consistently associated with major non-communicable diseases, including obesity [1]. Mechanistically, insufficient fiber intake disrupts lipid metabolism by impairing bile acid recycling, increasing cholesterol absorption, and altering gut microbiota composition, which in turn affects the production of short-chain fatty acids (SCFAs) [1,2,3,4,5]. Obese individuals often exhibit reduced microbial diversity and a lower abundance of butyrate-producing bacteria such as *Faecalibacterium*, *Roseburia*, and *Eubacterium*, together with an enrichment of taxa linked to systemic inflammation and adiposity markers, including *Ruminococcus*, *Coprococcus*, and *Clostridium* [6,7,8]. These dysbiotic shifts impair SCFA production and exacerbate lipid dysregulation and chronic inflammation. Acetate, propionate, and butyrate—the main SCFAs—exert pleiotropic effects on host physiology: propionate regulates hepatic cholesterol synthesis and fatty acid oxidation; butyrate serves as a major energy source for colonocytes and modulates inflammatory responses through epigenetic regulation; and acetate contributes to systemic lipid and glucose homeostasis [6,9]. Collectively, SCFAs constitute a mechanistic link between fiber intake and the attenuation of obesity-associated metabolic dysfunction [10]. Dysbiosis and reduced SCFA production have also been associated with comorbidities such as metabolic associated fatty liver disease (MAFLD) [5].

The relationship between dietary fiber, gut microbiota, and obesity is increasingly recognized as a key determinant of metabolic health [11]. Transcriptomic sequencing approaches have been widely employed to elucidate the molecular mechanisms through which dietary fiber and SCFAs modulate host metabolism. RNA-seq studies in liver tissue from obese models fed high-fiber diets have revealed differential expression of genes involved in lipid metabolism, bile acid signaling, and inflammatory pathways [12]. Integrative omics analyses have further demonstrated that SCFAs influence hepatic gene expression, providing mechanistic insight into how fiber-derived metabolites regulate host energy balance and systemic inflammation [9].

Together, the lack of fiber intake and microbiota dysbiosis may contribute to systemic inflammation and metabolic imbalance [13]. Consumption of fiber-rich fruits such as mango has been suggested to support metabolic health and favorably modulate the gut microbiota [14]. However, fruit intake remains notably low, e.g., in Mexico only one in four children meets the recommended daily intake of fruits and vegetables [15]. Recent research has emphasized the need to increase dietary fiber availability while reducing agro-industrial waste [8], highlighting that bioactive compounds present in fruit by-products such as mango peel can still be effectively utilized [16]. In this context, mango bagasse—a by-product of the juice industry—has been successfully incorporated into functional confectionery due to its content of gallic acid, mangiferin, quercetin, and dietary fiber [17]. Luzardo-Ocampo et al. [18] reported that a confectionery containing mango bagasse promoted notable growth of *Lactobacillus helveticus* and other *Lactobacillus* species, attributed both to lactic fermentation of hexoses and to the prebiotic activity of phenolics such as gallic acid, (+)-catechin, mangiferin, and quercetin.

Given that low fiber intake contributes to obesity, gut microbiota dysbiosis, reduced SCFA production, and alterations in hepatic lipid metabolism, and considering that fruit by-products such as mango bagasse and peel offer a sustainable source of dietary fiber and bioactive compounds, incorporating these materials into commonly consumed foods represents a promising strategy to support metabolic health. Previously, a confection was formulated using mango by-product fiber and tested in vitro, yielding promising results regarding its potential prebiotic activity. Its fermentability suggested an interesting short-chain fatty acid (SCFA) profile that warranted evaluation in an in vivo model to determine its metabolic effects [19]. Our previous work demonstrated that a mango bagasse- and peel-based confectionery (MC) modified the abundance of obesogenic gut microbial species and was associated with improvements in lipid-related parameters in rats [8]. However, whether these microbiota shifts translate into changes in hepatic gene expression remains unclear. To address this gap, the present study investigated whether MC consumption modulates gut microbiota, SCFA profiles, and hepatic transcriptomic responses in Wistar rats fed either a standard diet (STD) or a high-fat diet (HFD).

## 2. Materials and Methods

### 2.1. Confectionery Procurement

The MC was prepared and supplied by the Chemistry and Carbohydrate Functionality Laboratory at Universidad Autónoma de Querétaro (UAQ), Mexico. The composition of the MC was determined using AOAC official methods [20], resulting in the following proximate composition: moisture, 48%; insoluble fiber, 13.7%; soluble fiber, 10.7%; protein, 11.4%; carbohydrates, 2.7%; ash, 0.8%; and fat, <0.1%.

### 2.2. Animal Treatment

#### 2.2.1. Experimental Design and Diet

The animal experimental procedures were reviewed and approved on 24 May 2021 by the Ethical Committee of UAQ (approval ID: CBQ21/015) and conducted in accordance with the Mexican standard NOM-062-ZOO-1999 [21] and the National Institutes of Health (NIH) Guidelines for the Care and Use of Laboratory Animals. Twenty-four male Wistar rats (4 weeks old, average weight 119 g) were obtained from the Instituto de Neurobiología at Universidad Nacional Autónoma de México (UNAM), Campus Juriquilla. All animals were included in the experiment; no exclusions were made. Only male rats were used to minimize hormonal variability. Animals were housed individually under controlled environmental conditions (temperature, 22 ± 4 °C; relative humidity, 50 ± 15%; 12 h light/dark cycle).

Animals were randomly assigned to four groups (n = 6): standard diet (STD), standard diet supplemented with MC (MC-STD), high-fat diet (HFD; 60% energy from fat), and high-fat diet supplemented with MC (MC-HFD). The standard diet (Laboratory Rodent Diet 5001, LabDiet, Land O’Lakes^®^, Inc., St. Louis, MO, USA) contained 5.3% total fiber, whereas the high-fat diet (DIO Rodent Purified Diet with 60% Energy from Fat—Blue 58Y1, TestDiet^®^, Land O’Lakes, Inc., CA, USA) contained 6.5% total fiber. All dietary treatments were administered for 11 consecutive weeks, representing an intermediate experimental period (6–15 weeks) commonly used in this type of study [22,23,24]. The sample size (n = 6 per group) was selected based on reports from previous similar studies [25,26].

#### 2.2.2. Treatment

MC was administered daily and adjusted to provide 5.9 g of fiber per 1000 kcal consumed, in accordance with the daily intake recommendations [27] and based on the average fiber consumption reported for Mexican children [28]. Administration consisted of allowing the animals to consume the entire MC portion without access to food for up to 30 min in the morning. Food and water were available ad libitum throughout the study. After the treatment period, animals were euthanized following a 12 h fasting period using controlled CO_2_ exposure and cardiac puncture.

#### 2.2.3. Measurement of Feed Intake, Body Composition, and Sample Collection

During the treatment period, food intake was recorded daily, and body weight was measured twice per week to monitor the animals’ feeding behavior and growth. At the end of the intervention, the final body weight and body length were recorded. The liver was then weighed and sectioned; one portion was snap-frozen in liquid nitrogen and stored at −20 °C until cholesterol extraction, whereas another portion was placed in RNAlater solution (Thermo Fisher Scientific, Waltham, MA, USA), snap-frozen in liquid nitrogen, and stored at −20 °C until RNA extraction.

Liver lipid extraction was performed following the Folch method [29]. Cholesterol quantification was conducted by gas chromatography–mass spectrometry (GC–MS; 7890A GC coupled with 5975C inert MSD with Triple-Axis Detector, Agilent Technologies, Santa Clara, CA, USA) using a cholesterol calibration curve (6–25 mg/mL) (Product No. C8503, Lot #SLBC5917V; ≥92.5% purity by GC; Sigma-Aldrich, St. Louis, MO, USA). A 2 µL aliquot was injected with a 2:1 split into a DB-17HT capillary column (30 m × 0.25 mm i.d., 0.15 µm film thickness; Agilent Technologies, Santa Clara, CA, USA), using helium as the carrier gas (1 mL/min). The initial oven temperature was set to 276 °C (held for 4 min), increased at 8 °C/min to 300 °C, and held for 5 min, for a total run time of 19 min.

### 2.3. Gut Microbiota and SCFA Analyses

#### 2.3.1. Sample Collection and DNA Extraction

Gut content was collected from the cecum immediately after euthanasia. Samples were snap-frozen in liquid nitrogen and stored at −80 °C until analysis. Genomic DNA was extracted using a Genomic DNA Extraction Kit (Promega Corporation, Madison, WI, USA) according to the manufacturer’s instructions. DNA concentration was determined using the Qubit DNA BR Assay Kit (Invitrogen, Carlsbad, CA, USA), and purity was assessed with a NanoDrop 1000 spectrophotometer (Thermo Fisher Scientific, Wilmington, DE, USA). The extracted DNA had an average concentration of 108.93 ± 77.02 ng/µL and a purity ratio of 2.009 ± 0.109 at A260/280.

#### 2.3.2. Library Preparation and 16S Sequencing

Primers targeting the variable V3 and V4 regions of the 16SrRNA gene that included the adapter regions compatible with the Nextera XT Index kit (Illumina, San Diego, CA, USA) were used to prepare the sequencing libraries. Briefly, for each sample, 5 ng of gDNA mixed with 0.2 µM of Forward Primer (16S Amplicon PCR Forward Primer = 5′-TCGTCGGCAGCGTCAGATGTGTATAAGAGACAGCCTACGGGNGGCWGCAG-3′), 0.2 µM of Reverse Primer (16S Amplicon PCR Reverse Primer = 5′-GTCTCGTGGGCTCGGAGATGTGTATAAGAGACAGGACTACHVGGGTATCTAATCC-3′), and 1X PrimeSTAR HS DNA Polymerase (Takara, Kusatsu, Shiga, Japan) were amplified using the following PCR method: 1 min at 98 °C, 25 cycles of 30 s at 98 °C, 30 s at 55 °C and 30 s at 72 °C and a final extension of 2 min at 72 °C. PCR products were purified using AMPure XP beads (Beckman Coulter Inc., Brea, CA, USA) in a 0.8:1 bead:sample volume ratio. Index sequences were attached to each library by PCR. For each library, 1 µL of purified amplification product from the V3–V4 PCR was mixed with 1.5 µL of each individual index, 3.5 µL of nuclease-free water and 7.5 µL of PrimeSTAR HS DNA Polymerase. Index PCR method was: 1 min at 98 °C, 11 cycles of 20 s at 98 °C, 20 s at 55 °C and 20 s at 72 °C and a final extension of 2 min at 72 °C. Indexed libraries were purified using AMPure XP beads in a 1.12:1 bead:sample volume ratio. Libraries were quantified with Qubit dsDNA HS Assay Kit (Invitrogen, Carlsbad, CA, USA), their size was analyzed in a QSep 400 (BiOptic, New Taipei City, Taiwan), and sequencing was performed in a MiSeq using the MiSeq Reagent kit V3 (Illumina, San Diego, CA, USA) in a 301 bp pair-end reads configuration.

##### Quality and Preprocessing Analysis

A quality assessment of the 16S rRNA libraries was performed with FastQC (Babraham Bioinformatics, Cambridge, UK) to determine the sequencing quality (Supplementary File S1). All raw sequences passed the initial quality filter. Adapters were removed, and a quality and length filter were performed using Trimmomatic 0.40 [30]. For the Taxonomic Classification, Kraken2 [31] was used to analyze the taxonomic community compositions of Wistar rat microbiota, using the previously processed reads as input with a confidence threshold of 0.5. The vegan package was used to calculate the diversity indexes, while ggplot2 was used to produce the stacked bar plots in R (version 3.4.1, R Core Team, 2019) [32].

#### 2.3.3. Quantification of SCFAs

Frozen samples were lyophilized to quantify SCFAs by GC-MS (model 7890A coupled with model 5975C inert MSD with Triple-Axis Detector, Agilent Technologies), following the methodology of Figueroa L. et al. [33]. Before injection, 0.0200 g of gut content was diluted in 180 µL of HPLC-grade water and passed through a 0.45 µm pore size filter. A volume of 0.5 mL was injected with a 0.5:1 split into an HP-FFAP separation column (30 m, i.d. 0.250 mm, film 0.25 µm) (Agilent Technologies, Santa Clara, CA, USA), using helium as the carrier gas (1 µL/min). The injection temperature was set to 240 °C, the initial column temperature was 110 °C (held for 1 min), then ramped at 15 °C/min to 185 °C, with a detector temperature of 240 °C; the total run time was 11 min. The calibration curve (0.005–10 mmol) was prepared using Volatile Free Acid Mix (Sig-ma-Aldrich, St. Louis, MO, USA) to quantify acetate, propionate, and butyrate.

### 2.4. Gene Expression

#### 2.4.1. RNA Extraction, Library Preparation, and Sequencing

Total RNA was isolated from liver samples using the RNeasy Mini Kit (Qiagen, Hilden, Germany) following the manufacturer’s instructions. Tissues were lysed and homogenized in 600 µL of lysis buffer using a FastPrep system (MP Biomedicals, Irvine, CA, USA) at 5.5 m s^−1^ for 45 s. RNA concentration was determined with the Qubit RNA HS Assay Kit (Thermo Fisher Scientific, USA), integrity was assessed with a QSep 400 system (BiOptic, Taiwan), and purity was evaluated using a NanoDrop 1000 spectrophotometer (Thermo Fisher Scientific, Wilmington, DE, USA). Sequencing libraries were constructed with the TruSeq Stranded Total RNA Library Prep Kit with Ribo-Zero Gold (Illumina, San Diego, CA, USA), adjusting fragmentation times according to the RNA integrity number (RIN). Libraries were quantified using the Qubit dsDNA HS Assay Kit (Invitrogen, USA), and fragment size distribution was verified with the QSep 400 system. Sequencing was performed on a NovaSeq 6000 platform (Illumina) using a 100 bp paired-end configuration.

#### 2.4.2. Quality and Preprocessing Analysis

The quality of the raw reads was assessed using FastQC [34]. Since all quality metrics were satisfactory, the reads were aligned to the reference genome using STAR software version: 2.7.10b [35]. Alignment results were summarized in a gene-level read count matrix, which was subsequently used for differential expression analysis.

#### 2.4.3. Differential Expression and Enrichment Analysis

Differential gene expression was analyzed using the DESeq2 package in R [36]. Statistical significance between experimental conditions was determined by applying a false discovery rate (FDR) < 0.05 and an absolute log_2_ fold change > 1.0. The resulting gene lists were subsequently analyzed with iPathwayGuide (Advaita Bioinformatics, Ann Arbor, MI, USA) to identify significantly enriched biological pathways.

#### 2.4.4. qPCR Analysis

Complementary DNA (cDNA) was synthesized from total RNA using the High-Capacity cDNA Reverse Transcription Kit (Applied Biosystems, Thermo Fisher Scientific, Carlsbad, CA, USA), following the manufacturer’s instructions. Quantitative PCR (qPCR) was performed with gene-specific primers for Hmgcs1 (forward: 5′-CACAGCAACCAGGGCTCTGTGG-3′; reverse: 5′-TCAGCACCACAGCAAGCTTCCG-3′) and Cyp4a14 (forward: 5′-ACGAGGAAGGCTCAGCTGCAGA-3′; reverse: 5′-ACCTCTGCACGCAGGTCCTCAT-3′), which were designed using Benchling (San Francisco, CA, USA) and synthesized by T4 Oligo (Irapuato, Gto, Mexico). Reactions were carried out using SYBR Green PCR Master Mix (Applied Biosystems, Carlsbad, CA, USA) on a StepOne™ Real-Time PCR System (Applied Biosystems, Carlsbad, CA, USA), as described by Melgar-Rojas et al. [37]. Relative gene expression was quantified using the 2^−ΔΔCt^ method [38].

### 2.5. Statistical Analysis

Data on eating behavior, body composition, species relative abundances (RAs), and SCFAs were expressed as mean ± standard error (SE). Significant differences among groups were evaluated by one-way ANOVA followed by Tukey–Kramer post hoc test (*p* < 0.05) using JMP^®^ Pro 14.0.0 (SAS Institute Inc., Cary, NC, USA). Principal component analysis (PCA), as well as dispersion, distribution, and correlation matrices, together with microbiota diversity and composition analyses, were performed in R (version 4.2.1) using the vegan, ggvegan, MASS, and psych packages. Pearson correlations were calculated to explore patterns among gut microbiota species, fiber intake, and metabolic variables, and were used to describe trends within the dataset.

## 3. Results

### 3.1. Animal Growth

Total feed intake differed according to diet type (Figure S1a), with higher values observed in the STD groups (*p* < 0.0001). Total fiber intake was greatest in the MC-STD group (*p* < 0.0001, Figure S1b), followed by the STD and MC-HFD groups (*p* = 0.1229), while the HFD group showed the lowest fiber intake (*p* < 0.0001). Final body weight did not differ within the STD groups (*p* = 0.7077) or within the HFD groups (*p* = 0.9363, Figure S1c); however, both HFD groups had significantly higher final body weight compared with the STD groups (*p* < 0.05). Weight gain was lowest in the STD group (*p* < 0.05), intermediate in the MC-groups (*p* = 0.6158), and highest in the HFD groups (*p* < 0.05, Figure S1d). Feed efficiency, calculated from weight gain and feed intake, was also greater in the HFD groups (*p* < 0.05, Figure S1e). Liver weight was similar across groups (*p* > 0.05, Figure S1f), but total liver cholesterol content was elevated in the HFD groups (*p* < 0.005, Figure S1g).

**Figure 1:**
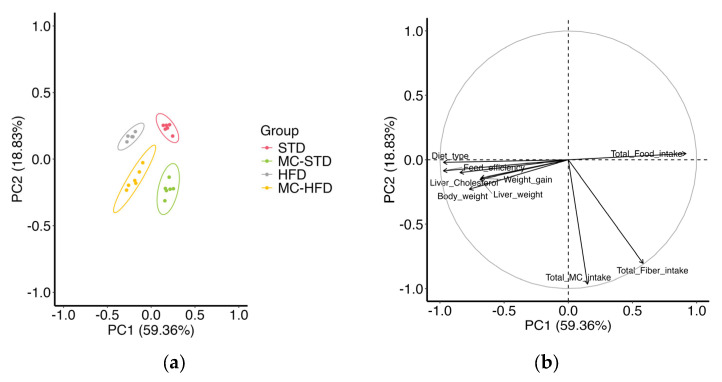
PCA of eating behavior and body composition variables. (**a**) PCA score plot showing clustering of STD, MC-STD, HFD, and MC-HFD groups. (**b**) PCA scatter plot illustrating the contribution of diet type, feed efficiency, body and liver variables, and fiber and MC intake.

Although some variables did not differ markedly, PCA integrating eating behavior and body composition revealed distinct clustering by diet (Figure 1a), with PC1 and PC2 together explaining 78% of the variance. The strongest contributors were total fiber intake and feed efficiency, followed by MC intake, which was positively associated with fiber intake (Table S1). Accordingly, MC-supplemented groups clustered toward the lower section of the plot, HFD groups to the left, and STD groups mainly to the right, reflecting feed efficiency distribution (Figure 1a,b). Body weight, weight gain, liver weight, and liver cholesterol were associated with diet type and feed efficiency (Figure 1b), overlapping with the HFD groups.

### 3.2. Gut Microbiota and SCFAs

#### 3.2.1. Gut Microbiota Diversity and Taxonomic Composition

**Figure 2:**
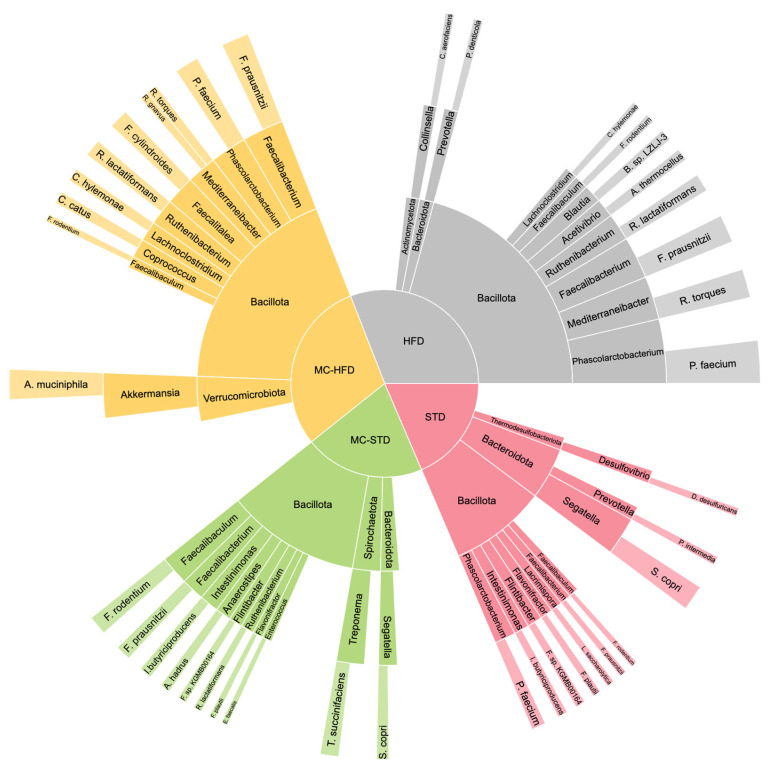
Sunburst chart showing gut microbiota taxa across dietary groups (STD, MC-STD, HFD, and MC-HFD). Taxa are represented hierarchically at three taxonomic levels—phylum, genus, and species—based on the top 10 most abundant species.

Comparisons of the alpha diversity of the gut microbiota at the phylum, genus, and species levels revealed significant differences depending on diet type (*p* < 0.05); however, no effect was observed for MC supplementation (Figure S2a–c). The top 10 RAs for each group were obtained at the phylum, genus, and species levels, showing differences in intestinal microbiota composition among groups (Figure S2d–f). At the phylum level, Bacillota was dominant across all groups (Figure 2). The second most abundant phylum differed: Bacteroidota in STD and HFD, Spirochaetota in MC-STD, and Verrucomicrobiota in MC-HFD, the latter including *Akkermansia muciniphila*, detected exclusively at high RA in MC-HFD.

MC-supplemented groups presented *Faecalibacterium*, *Ruthenibacterium*, and *Faecalibaculum*, sharing with STD *Phascolarctobacterium*, *Intestimonas*, *Flintibacter*, *Flavonifractor*, and *Segatella*. HFD uniquely presented *Acetivibrio*, *Blautia*, and *Collinsella*, while also sharing *Prevotella*, *Lachnoclostridium*, *Mediterraneibacter*, and others with other groups. At the species level, *A. muciniphila* was most abundant in MC-HFD, followed by *Faecalibacterium prausnitzii* (third in MC-STD and HFD; eighth in STD). *Faecalibaculum rodentium* was detected in all groups, *Phascolarctobacterium faecium* in all except MC-STD, and *Ruthenibacterium lactatiformans* in MC-supplemented and HFD but absent in STD. *Ruminococcus torques* and *Clostridium hylemonae* were present in both HFD groups, whereas *Ruminococcus gnavus, Faecalitalea cylindroides*, and *Coprococcus catus* were detected only in the MC-HFD group. HFD exclusively contained *Collinsella aerofaciens*, *Blautia* sp. LZLJ-3, and *Acetivibrio thermocellus*. STD groups uniquely showed *Flavonifractor plautii, Segatella copri*, and *Flintibacter* sp. KGMB00164, whereas MC-STD uniquely contained *Intestimonas butyriciproducens*, *Anaerostipes hadrus*, *Enterococcus faecalis,* and *Treponema succinifaciens*. Although *Prevotella* occurred in both STD and HFD, the species differed: *P. intermedia* in STD and *P. denticola* in HFD.

#### 3.2.2. Gut Microbiota Correlations with Fiber Intake and Body Composition

The PCA integrating gut microbiota composition with eating behavior and body composition variables is shown in Figure S3a, with PC1 and PC2 together explaining 46% of the variance. MC-supplemented groups were positioned toward the upper region of the plot, coinciding with fiber and MC intake (Figure S3a,b), whereas control groups (STD and HFD) appeared toward the lower region. HFD groups tended to cluster on the right side, in association with feed efficiency, with the HFD group in particular overlapping with body weight, liver weight, and liver cholesterol. Clustering patterns were primarily driven by diet type and feed efficiency (Table S2). The species most strongly represented in PC1 and PC2 were *I. butyriciproducens*, *S. copri*, *L. saccharolytica*, *C. catus*, *E. faecalis*, *P. faecium*, and *F.* sp. KGMB00164 (Table S2). Among these, *I. butyriciproducens*, *E. faecalis*, and *C. catus* were associated with the region containing MC-supplemented groups, whereas *S. copri*, *L. saccharolytica*, and *F*. sp. KGMB00164 were located in the area corresponding to the STD group. Notably, *P. faecium* was found in the region associated with the HFD group, showing a positive relationship with body weight, feed efficiency, and liver cholesterol, and a negative relationship with fiber intake.

**Figure 3:**
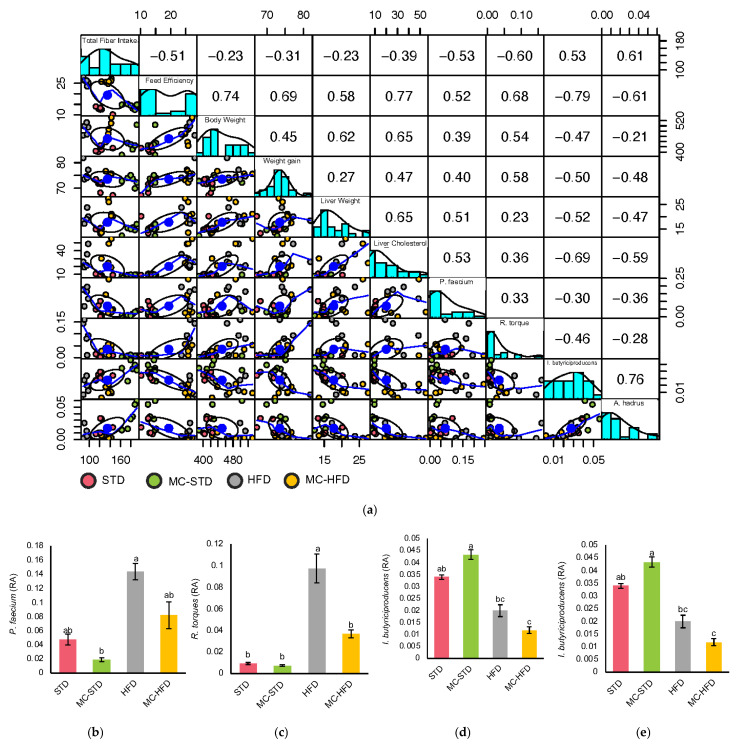
Gut microbiota species correlated with fiber intake and metabolic variables. (**a**) Scatterplot matrix showing distributions and pairwise correlations of gut microbiota species associated with fiber intake and metabolic variables; RAs of (**b**) *P. faecium*; (**c**) *R. torques*; (**d**) *I. butyriciproducens*; and (**e**) *A. hadrus*. Significant differences (*p* < 0.05) were determined by one-way ANOVA followed by Tukey–Kramer’s post hoc test and are indicated by different letters.

Scatter matrix correlations revealed four species linked to fiber intake: *P. faecium*, *R. torques*, *I. butyriciproducens*, and *A. hadrus* (Figure 3a). The correlation of fiber intake with each of these microorganisms showed the same clustering pattern, in which the MC-STD group was positioned on the left, the STD and MC-HFD groups in the center, while the HFD group appeared on the right (Figure 3a). Regarding the correlations of weight gain, *P. faecium* and *R. torques* clustered with the STD groups in the lower left, the MC-HFD group in the center, and the HFD group in the upper area, while correlations with *I. butyriciproducens* and *A. hadrus* showed less defined clustering. Overall, *P. faecium* and *R. torques* were negatively correlated with fiber intake and positively correlated with body variables such as weight gain and liver cholesterol. In contrast, *I. butyriciproducens* and *A. hadrus* were positively correlated with fiber intake and negatively correlated with body variables. Although their RAs did not differ significantly between supplemented groups and their respective controls (*p* > 0.2000), certain patterns were evident: for *P. faecium* and *R. torques*, RA was higher in the control groups compared with the MC-supplemented groups (Figure 3b,c). However, for *I. butyriciproducens* and *A. hadrus*, the highest RA was observed in the MC-STD group, followed by the control groups, and lowest in the MC-HFD group (Figure 3d,e).

#### 3.2.3. SCFAs Concentrations

**Figure 4:**
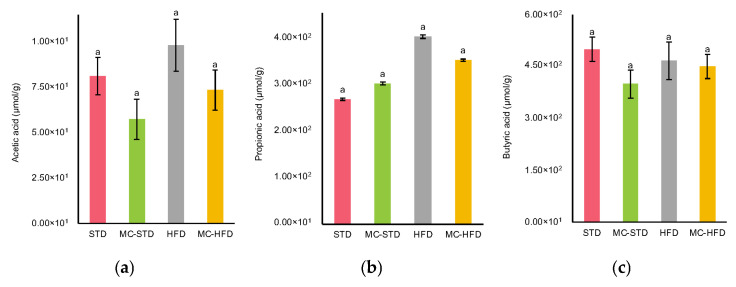
SCFA concentrations in gut content. (**a**) Acetate; (**b**) propionate; (**c**) butyrate. Data are expressed as mean ± SEM. Significant differences (*p* < 0.05) were determined by one-way ANOVA followed by Tukey–Kramer post hoc test and are indicated by different letters.

Acetate was the most abundant SCFA across all groups (Figure 4a), followed by butyrate (Figure 4c), whereas propionate showed the lowest concentrations (Figure 4b). MC supplementation did not significantly affect SCFA production, as no differences were observed between groups. However, acetate concentrations tended to be higher in the control groups, while propionate concentrations tended to be higher in the HFD groups. In contrast, butyrate did not exhibit a clear trend among the dietary treatments.

### 3.3. Gene Expression

#### 3.3.1. Transcriptomic Overview

**Figure 5:**
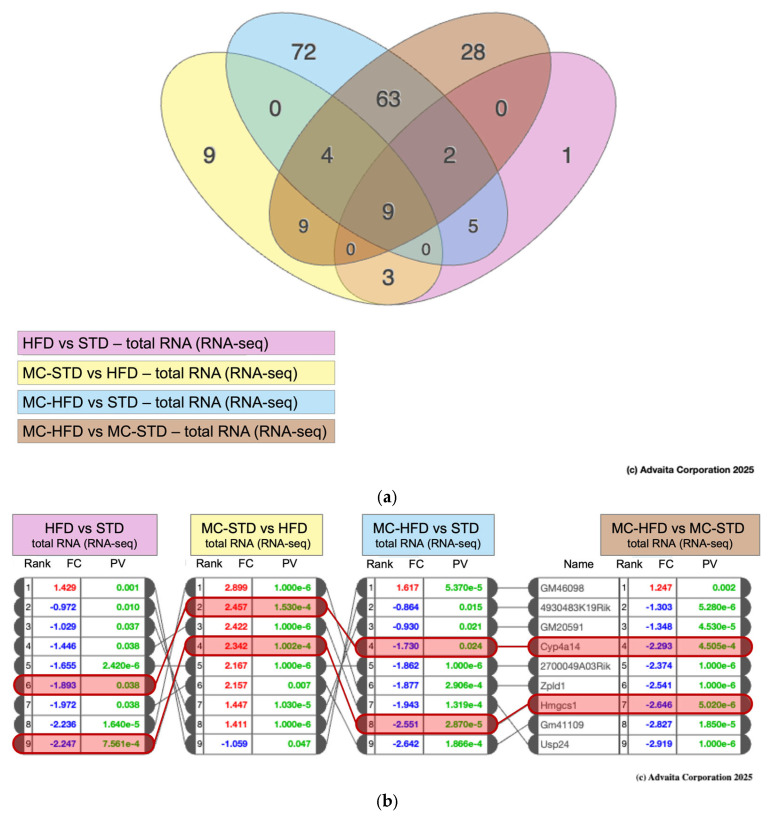
Transcriptomic analysis. (**a**) Venn diagram of differentially expressed genes across the contrasts HFD vs. STD, MC-STD vs. HFD, MC-HFD vs. STD, and MC-HFD vs. MC-STD; (**b**) consistently differentially expressed genes across contrasts, including Cyp4a14 and Hmgcs1, both involved in lipid metabolic pathways.

Hepatic transcriptomic profiling was conducted to evaluate diet- and fiber-related differences in lipid metabolism. A Venn diagram illustrates the significantly differentially expressed genes (DEGs) across group contrasts (Figure 5a). Although all pairwise comparisons were performed, only those showing significant differential expression are reported (*p* < 0.05): HFD vs. STD, MC-STD vs. HFD, MC-HFD vs. STD, and MC-HFD vs. MC-STD. Across these contrasts, nine genes were consistently differentially expressed, including cytochrome P450 family 4 subfamily A polypeptide 14 (Cyp4a14) and 3-hydroxy-3-methylglutaryl-CoA synthase 1 (Hmgcs1), both involved in lipid metabolic pathways (Figure 5b).

#### 3.3.2. Pathway Analysis and Differential Gene Expression

**Figure 6:**
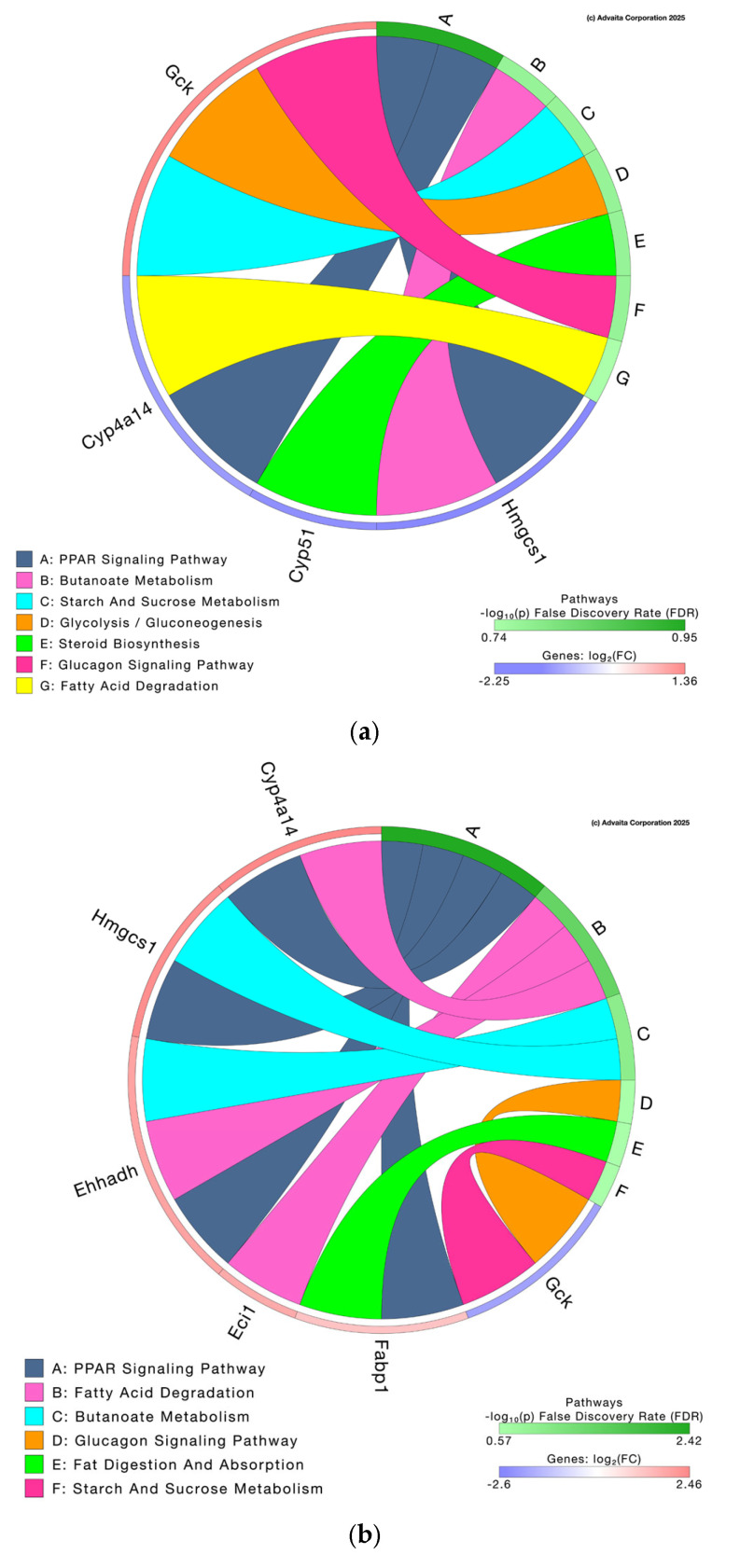

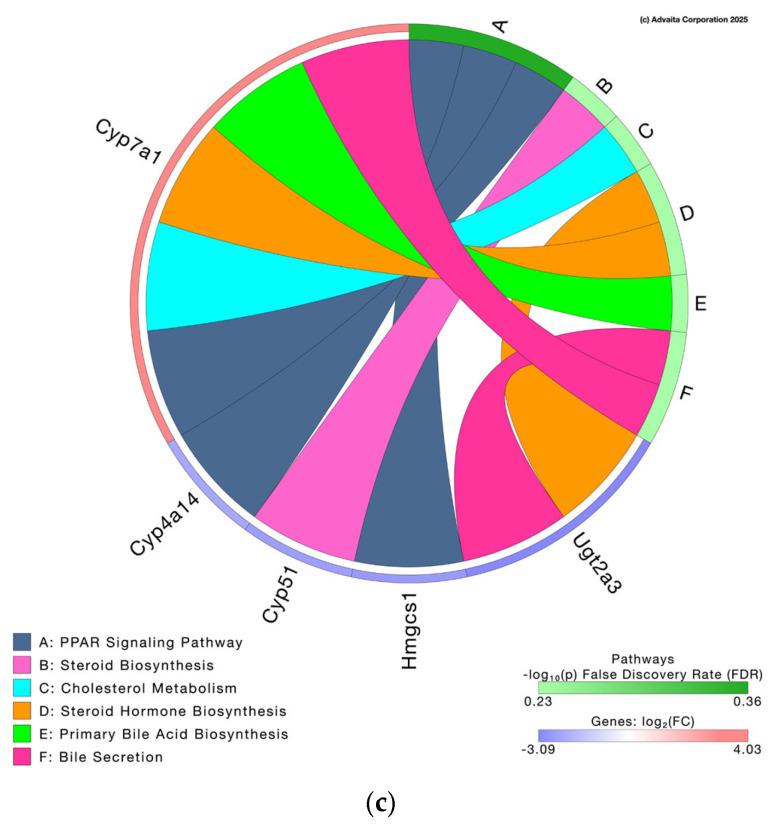
Pathways impacted by differentially expressed genes. (**a**) HFD vs. STD; (**b**) MC-STD vs. HFD; (**c**) MC-HFD vs. MC-STD.

When analyzed individually, the HFD vs. STD contrast included 10,162 genes, of which 20 were differentially expressed; the MC-STD vs. HFD contrast included 9642 genes with 34 differentially expressed; and both the MC-HFD vs. STD and MC-HFD vs. MC-STD contrasts included 11,719 genes, each with 155 differentially expressed (Figure S4a–d). The impact of differentially expressed genes on lipid-related pathways was then examined. In the HFD vs. STD contrast, four differentially expressed genes affected seven metabolic pathways (Figure 6a). Notably, the peroxisome proliferator-activated receptor (PPAR) signaling pathway was impacted through the downregulation of Cyp4a14 and Hmgcs1, which also influenced fatty acid degradation and butanoate metabolism, respectively. The MC-STD vs. HFD contrast revealed six pathways affected by six differentially expressed genes (Figure 6b). Again, the PPAR signaling pathway was affected, in this case through the upregulation of Cyp4a14, Hmgcs1, enoyl-CoA hydratase and 3-hydroxyacyl-CoA dehydrogenase (Ehhadh), and fatty acid binding protein 1 (Fabp1). The fatty acid degradation pathway was influenced by the upregulation of Cyp4a14, Ehhadh, and enoyl-CoA delta isomerase 1 (Eci1), whereas Fabp1 impacted fat degradation. In contrast, the MC-HFD vs. STD comparison showed no changes in differential expression (thus, we did not add this analysis to the article). Finally, the MC-HFD vs. MC-STD contrast showed six pathways affected by five differentially expressed genes (Figure 6c). In this comparison, PPAR signaling was influenced by the upregulation of cytochrome P450 family 7 subfamily A member 1 (Cyp7a1) and the downregulation of Cyp4a14 and Hmgcs1. Cyp7a1 also impacted cholesterol metabolism, primary bile acid biosynthesis, and bile secretion, the latter additionally affected by the downregulation of UDP-glucuronosyltransferase family 2 member A3 (Ugt2a3).

**Figure 7:**
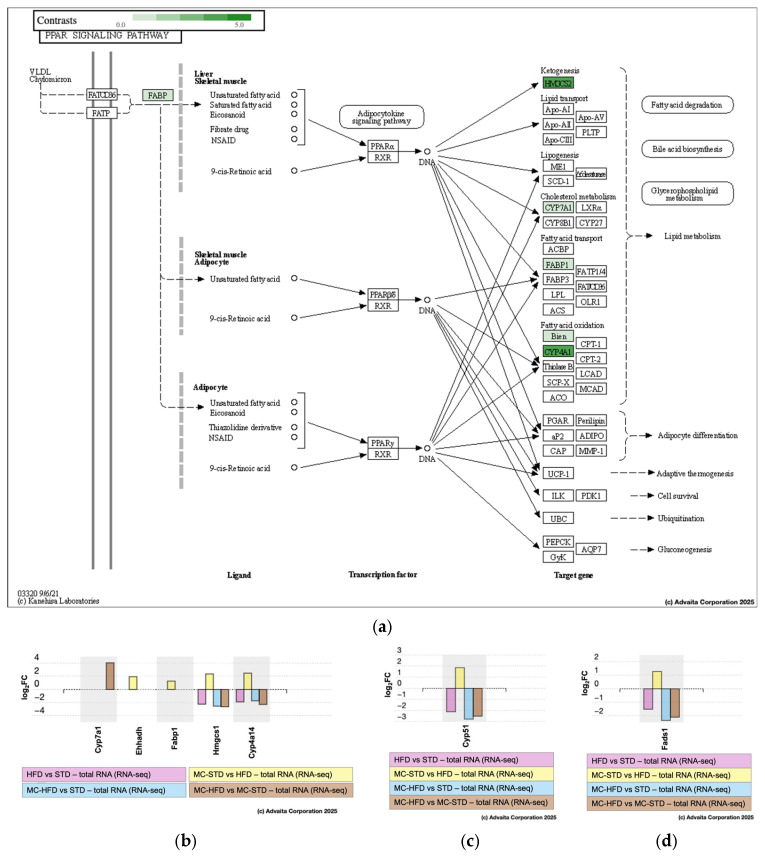
Differentially expressed genes related to PPAR signaling. (**a**) Differentially expressed genes associated with the PPAR signaling pathway; log2 fold change of (**b**) Cyp4a14 and Hmgcs1; (**c**) Cyp51; and (**d**) Fads1 across experimental contrasts.

Overall, the most consistently impacted pathway across contrasts was PPAR signaling (Figure 7a), particularly regarding lipid metabolism. Hmgcs1 was downregulated in HFD vs. STD (Log2FC = −2.247, *p* = 0.0008), MC-HFD vs. STD (Log2FC = −2.551, *p* < 0.0001), and MC-HFD vs. MC-STD (Log2FC = −2.646, *p* < 0.0001), but upregulated in MC-STD vs. HFD (Log2FC = 2.342, *p* = 0.0001) (Figure 7b). Cyp4a14 showed a similar pattern (Figure 7b), being downregulated in HFD vs. STD (Log2FC = −1.893, *p* = 0.0380), MC-HFD vs. STD (Log2FC = −1.730, *p* = 0.0240), and MC-HFD vs. MC-STD (Log2FC = −2.293, *p* = 0.0004), while upregulated in MC-STD vs. HFD (Log2FC = 2.457, *p* = 0.0001). Since these genes were the most relevant, their differential expression was validated by qPCR, showing a pattern consistent with the transcriptomic analysis. In the HFD vs. STD, MC-HFD vs. STD, and MC-HFD vs. MC-STD contrasts, both Hmgcs1 and Cyp4a14 were decreased (Figure S5a,b).

PPAR signaling was also influenced by Fabp1 (fatty acid transport) and Ehhadh (fatty acid oxidation), both upregulated in MC-STD vs. HFD (Log2FC = 1.240, *p* = 0.0400 and Log2FC = 1.909, *p* = 0.0490, respectively) (Figure 7a,b). Conversely, the MC-HFD vs. MC-STD contrast showed upregulation of Cyp7a1 (Log2FC = 4.032, *p* = 0.0050), impacting cholesterol metabolism (Figure 7a,b). In addition to genes involved in PPAR signaling, cytochrome P450 family 51 (Cyp51) and fatty acid desaturase 1 (Fads1), also linked to lipid metabolism, were evaluated for differential expression. Both exhibited expression patterns similar to Hmgcs1 and Cyp4a14. They were downregulated in HFD vs. STD (Cyp51: Log2FC = −2.125, *p* = 0.0380; Fads1: Log2FC = −1.516, *p* = 0.5510), MC-HFD vs. STD (Cyp51: Log2FC = −2.791, *p* = 0.0003; Fads1: Log2FC = −2.349, *p* = 0.0130), and MC-HFD vs. MC-STD (Cyp51: Log2FC = −2.520, *p* = 0.0020; Fads1: Log2FC = −2.105, *p* = 0.0430), but upregulated in MC-STD vs. HFD (Cyp51: Log2FC = 1.853, *p* = 0.1040; Fads1: Log2FC = 1.272, *p* = 1.0000) (Figure 7c,d).

## 4. Discussion

Fruit-derived fibers, rich in soluble and antioxidant fiber, have been linked to improved gut health and microbiota composition [3,14,39]. Since the gut microbiota and its metabolites can influence hepatic gene expression through the gut–liver axis, their modulation has been proposed as a therapeutic target for metabolic disorders [40,41,42,43]. Strategies to modulate the microbiota range from fruit pulp consumption to supplementation with beta-glucans, pectins, and phenolic compounds [2,3,11,14,44,45], and the use of agribusiness by-products is emerging as a sustainable alternative. In line with this, our recent study reported that a mango by-product–based confectionery may help prevented liver fat accumulation and tended to reduce plasma cholesterol in rats via the gut–liver axis [8].

In this study, we explored the effect of a confectionery enriched with fiber from mango juice by-products—extruded bagasse and peel—on gut microbiota, SCFAs and hepatic transcriptome. The MC was provided to Wistar rats fed either an STD or HFD at a dosage designed to meet the recommended daily fiber intake for Mexican children based on national average [28]. No significant differences in weight gain were observed between the MC groups and their respective controls, which is consistent with previous reports in mice supplemented with isolated pectin [2]. where doses below 10% did not produce changes in weight gain. However, unlike isolated fibers, the intervention in our study consisted of a whole-food product. For this reason, the dose provided did not reach the >10% level commonly used in extract-based studies, as it was intentionally formulated to reflect a realistic dietary intake rather than a concentrated supplementation. Despite this, PCA indicated that MC supplementation may have altered the dietary response of rats fed an HFD, a model resembling a Western-style diet. The MC-HFD group shifted away from the HFD cluster toward the MC-STD group, suggesting an influence of fiber intake even under a high-fat background. Moreover, fiber intake was associated with gut microbiota modulation, with MC-supplemented groups showing notable changes in their top 10 RAs profiles. In particular, *P. faecium* and *R. torques* showed a trend of lower RA in MC-supplemented groups. *P. faecium* is a common species in the human gut that produces acetate and propionate from succinate rather than from carbohydrates [46], and succinate generated by other bacteria has been linked to obesity [47]. *R. torques*, on the other hand, is a mucin-degrading bacterium associated with visceral fat and metabolic diseases such as MAFLD through the production of metabolites other than SCFAs [48,49]. Thus, tended to have a lower RA of both *P. faecium* and *R. torques* in the MC-supplemented groups may reflect a healthier microbiota profile, since for *P. faecium* it suggests fewer succinate-producing harmful microorganisms, whereas for *R. torques* it implies a reduced production of metabolites that could otherwise impair host metabolism.

Other microorganisms correlated with fiber intake were *I. butyriciproducens* and *A. hadrus*, both related to butyrate production [50,51]. Although both species were positively correlated with fiber intake, their RA in the MC-HFD group was not higher than in the HFD control, unlike the MC-STD group, which did show a higher trend relative to its control. This pattern is noteworthy because reports indicate that the behavior of certain butyrate-producing bacteria can vary depending on the host’s physiological or metabolic context. For example, in inflammatory bowel disease (IBD), *A. hadrus* has been associated with aggravated inflammation and increased body weight [50]. These species may also coexist with butyrate-consuming taxa or with microbes that generate metabolites that counteract the expected benefits of butyrate, and they may harbor genes that contribute to these alternative pathways [52]. Consequently, in some contexts—such as IBD or diabetes— butyrate producers do not consistently exert beneficial effects [50,52]. In this study, the absence of a higher RA of *I. butyriciproducens* and *A. hadrus* in the MC-HFD group may therefore reflect diet-specific interactions rather than a less favorable microbial profile. Notably, MC supplementation appeared to increase their RA in STD conditions while showing the opposite pattern in HFD conditions. Such contrasting outcomes have also been described in the literature for *A. hadrus*, suggesting that responses may depend on pre-existing host states or dietary environments. These observations highlight that microbial responses to fiber may vary according to the metabolic conditions in which fermentation occurs. Although our sample size limits definitive conclusions, this variability opens the possibility for new hypotheses regarding how microbial RAs may differentially influence host metabolism depending on baseline dietary or physiological context.

As mentioned above, the gut microbiota produces several metabolites, among which SCFAs—acetate, propionate, and butyrate—are the most studied due to their role in host metabolism, including the regulation of hepatic cholesterol synthesis [40,53]. Acetate and propionate can enter the circulation and be utilized by the liver in gluconeogenesis and lipogenesis [54], whereas butyrate primarily serves as an energy source for enterocytes [53]. Although more than 95% of SCFAs are normally absorbed by colonocytes [55], this process may vary with intestinal health and diet. Notably, high-fat diets have been reported to damage intestinal epithelial cells, potentially decreasing the absorption and utilization of SCFAs [56]. This mechanism may help contextualize our observation of a trend toward higher cecal SCFAs concentrations in HFD-fed groups—a finding that was not statistically significant and is not commonly reported in the literature. Although SCFA production has been associated with increased soluble fiber intake [55], in this study no significant differences were observed between groups, regardless of MC supplementation or diet type. This agrees with a review by Vinelli et al. [57], which reported that dietary fiber intake often alters gut microbiota composition without necessarily leading to changes in SCFA profiles. Given that most SCFAs are rapidly absorbed by colonocytes, their influence may be more evident in systemic metabolic outcomes than in cecal concentrations. SCFAs have been reported to modulate gene expression through AMPK activation, which can in turn lead to decreased PPAR activity in hepatic and adipose tissues [58]. Several molecules with antagonistic activity toward PPAR have been described; these include lipophilic acids of both synthetic and natural origin, such as the essential fatty acids docosahexaenoic and eicosapentaenoic acids [59], as well as SCFAs themselves [58]. Since the MC contains a very low lipid content (<0.1%), and the diet types did not differ in such composition, it is unlikely that the observed gene expression patterns were driven by fatty acids such as docosahexaenoic or eicosapentaenoic acids. Instead, the transcriptomic changes observed here are consistent with the SCFA-related regulatory mechanism: when PPAR-α activity decreases, the expression of PPAR-α–dependent genes involved in lipid metabolism, such as Cyp4a14, Hmgcs1, Cyp51, and Fads1, tends to decrease as well [54]. In this context, the downregulation of these genes in most contrasts aligns with this proposed mechanism; however, these associations should be interpreted as exploratory. The opposite pattern observed in the MC-STD vs. HFD contrast further highlights the complexity of these interactions.

Cyp4a14 is a homolog of human CYP4A, involved in hepatic fatty acid metabolism, and its upregulation has been associated with metabolic-associated fatty liver disease [60]. Hmgcs1 encodes a key enzyme in the cholesterol synthesis pathway, whose increased expression has been linked to elevated hepatic and plasma cholesterol [61]. Cyp51 participates in steroid biosynthesis, and its upregulation has been related to dyslipidemia [62]. Fads1 is involved in de novo lipogenesis in the liver, and its suppression has been shown to improve obesity-related biomarkers [63]. Thus, the downregulation of these genes observed in our study may reflect improvements in lipid metabolism, particularly cholesterol regulation, in HFD-fed rats supplemented with MC, which is consistent with our previous report showing a trend toward lower plasma total cholesterol was observed [8]. Moreover, these genes are part of the PPAR signaling pathway. The downregulation of PPAR, modulated by SCFAs, has been shown to improve obesity-related biomarkers induced by an HFD through a cascade that shifts hepatic metabolism from lipogenesis toward fatty acid oxidation [58]. In this context, the downregulation of Cyp4a14, Hmgcs1, Cyp51, and Fads1 observed in MC-supplemented groups could suggest that MC intake may exert favorable effects on hepatic lipid metabolism potentially by influencing PPAR signaling through SCFA-mediated related mechanisms. Notably, this is also in agreement with our earlier observation that MC supplementation was associated with protection against liver fat accumulation [8].

## 5. Conclusions

MC supplementation appeared to modulate the gut–liver axis by reducing obesogenic taxa, differentially influencing butyrate producers, and downregulating genes central to lipid metabolism. The RAs of obesogenic species such as *P. faecium* and *R. torques* showed a trend toward lower values with supplementation, whereas *I. butyriciproducens* and *A. hadrus* were differentially modulated depending on diet type and MC intake. Moreover, key hepatic genes involved in lipid metabolism, including *Cyp4a14*, *Hmgcs1*, *Cyp51*, and *Fads1*, were downregulated, potentially through mechanisms relate to SCFA–PPAR signaling. Overall, these findings highlight the potential of sustainable, fruit-derived fibers such as mango by-products to improve metabolic health under high-fat dietary conditions. This study was conducted in male Wistar rats; therefore, future research should examine potential sex-related differences and further investigate the molecular pathways underlying lipid metabolism.

## Data Availability

The data supporting the findings of this study are available in the Supplementary Materials.

## Supplementary Materials

The following supporting information can be downloaded at: https://www.mdpi.com/article/10.3390/nu17233780/s1, Figure S1: Eating behavior and body composition; Table S1: PCA cumulative loadings for variables associated with dietary intake and metabolic parameters; Figure S2: Alpha diversity and RAs of gut microbiota across taxonomic levels; Figure S3: PCA integrating gut microbiota, fiber intake, and body composition variables; Table S2: PCA cumulative loadings for variables associated with gut microbiota composition, dietary intake, and metabolic parameters; Figure S4: Volcano plots of differentially expressed genes across contrasts; and Figure S5: Differential gene expression across contrasts validated by qPCR. Supplementary File S1: FastQC—quality. Supplementary File S2: Raw relative abundances of all samples, at the Phylum, Genera, and Species level. Reference [64] is cited in the Supplementary Materials.

## Abbreviations

The following abbreviations are used in this manuscript:

AOAC	Association of Official Analytical Collaboration
Cyp4a14	Cytochrome P450 family 4 subfamily A polypeptide 14
Cyp51	Cytochrome P450 family 51
Cyp7a1	Cytochrome P450 family 7 subfamily A member 1
Eci1	Enoyl-CoA delta isomerase 1
Ehhadh	Enoyl-CoA hydratase and 3-hydroxyacyl-CoA dehydrogenase
Fabp1	Fatty acid binding protein 1
Fads1	Fatty acid desaturase 1
GC-MS	Gas chromatography–mass spectrometry
HFD	High-fat diet
Hmgcs1	3-hydroxy-3-methylglutaryl-CoA synthase 1
IBD	Inflammatory bowel disease
MAFLD	metabolic associated fatty liver disease
MC	Mango bagasse and peel confectionery
MC-HFD	High-fat diet supplemented with mango bagasse and peel confectionery
MC-STD	Standard diet supplemented with mango bagasse and peel confectionery
PCA	Principal component analysis
PPAR	Peroxisome proliferator-activated receptor
RAs	Relative abundances
SCFAs	Short-chain fatty acids
UAQ	Universidad Autónoma de Querétaro
Ugt2a3	UDP-glucuronosyltransferase family 2 member A3
