# Supplementary material for: Impact of Mango Bagasse and Peel Confectionery Rich in Dietary Fiber on Gut Microbiota, Metabolite Profiles, and Genetic Regulation in High-Fat-Diet-Fed Wistar Rats
Source: Nutrients. 2025 Dec 2;17(23):3780. doi: 10.3390/nu17233780 (PMC12694227; doi:10.3390/nu17233780)
Supplement: Supplementary file 1 [file nutrients-17-03780-s001.zip › nutrients-3998355-supplementary s1.pdf]

FASTQ Quality Check

Name: Dataset

Overall Results

Name	Per Base Sequence Quality	Per Sequence Quality Scores	Per Base Sequence Content	Per Sequence GC Content	Per Base N Content
M16SAur1_S1_L001_R1_001.fastq.gz	**PASS**	**PASS**	**FAIL**	**FAIL**	**PASS**
M16SAur1_S1_L001_R2_001.fastq.gz	**PASS**	**PASS**	**FAIL**	**FAIL**	**PASS**
M16SAur10_S10_L001_R2_001.fastq.gz	**FAIL**	**PASS**	**FAIL**	**WARNING**	**PASS**
M16SAur11_S11_L001_R2_001.fastq.gz	**FAIL**	**PASS**	**FAIL**	**FAIL**	**PASS**
M16SAur11_S11_L001_R1_001.fastq.gz	**PASS**	**PASS**	**FAIL**	**FAIL**	**PASS**
M16SAur10_S10_L001_R1_001.fastq.gz	**PASS**	**PASS**	**FAIL**	**WARNING**	**PASS**
M16SAur12_S12_L001_R2_001.fastq.gz	**FAIL**	**PASS**	**FAIL**	**FAIL**	**PASS**
M16SAur12_S12_L001_R1_001.fastq.gz	**PASS**	**PASS**	**FAIL**	**FAIL**	**PASS**
M16SAur13_S13_L001_R2_001.fastq.gz	**FAIL**	**PASS**	**FAIL**	**FAIL**	**PASS**
M16SAur13_S13_L001_R1_001.fastq.gz	**PASS**	**PASS**	**FAIL**	**FAIL**	**PASS**
M16SAur14_S14_L001_R2_001.fastq.gz	**FAIL**	**PASS**	**FAIL**	**FAIL**	**PASS**
M16SAur15_S15_L001_R2_001.fastq.gz	**FAIL**	**PASS**	**FAIL**	**FAIL**	**PASS**
M16SAur14_S14_L001_R1_001.fastq.gz	**PASS**	**PASS**	**FAIL**	**FAIL**	**PASS**
M16SAur15_S15_L001_R1_001.fastq.gz	**PASS**	**PASS**	**FAIL**	**FAIL**	**PASS**
M16SAur16_S16_L001_R2_001.fastq.gz	**FAIL**	**PASS**	**FAIL**	**FAIL**	**PASS**
M16SAur16_S16_L001_R1_001.fastq.gz	**PASS**	**PASS**	**FAIL**	**FAIL**	**PASS**
M16SAur17_S17_L001_R2_001.fastq.gz	**PASS**	**PASS**	**FAIL**	**WARNING**	**PASS**
M16SAur17_S17_L001_R1_001.fastq.gz	**PASS**	**PASS**	**FAIL**	**WARNING**	**PASS**
M16SAur18_S18_L001_R2_001.fastq.gz	**PASS**	**PASS**	**FAIL**	**FAIL**	**PASS**
M16SAur18_S18_L001_R1_001.fastq.gz	**PASS**	**PASS**	**FAIL**	**FAIL**	**PASS**
M16SAur19_S19_L001_R2_001.fastq.gz	**PASS**	**PASS**	**FAIL**	**FAIL**	**PASS**
M16SAur19_S19_L001_R1_001.fastq.gz	**PASS**	**PASS**	**FAIL**	**FAIL**	**PASS**
M16SAur2_S2_L001_R2_001.fastq.gz	**PASS**	**PASS**	**FAIL**	**FAIL**	**PASS**
M16SAur2_S2_L001_R1_001.fastq.gz	**PASS**	**PASS**	**FAIL**	**FAIL**	**PASS**
M16SAur20_S20_L001_R2_001.fastq.gz	**PASS**	**PASS**	**FAIL**	**FAIL**	**PASS**
M16SAur21_S21_L001_R2_001.fastq.gz	**WARNING**	**PASS**	**FAIL**	**FAIL**	**PASS**

M16SAur21_S21_L001_R1_001.fastq.gz	**PASS**	**PASS**	**FAIL**	**FAIL**	**PASS**
M16SAur20_S20_L001_R1_001.fastq.gz	**PASS**	**PASS**	**FAIL**	**FAIL**	**PASS**
M16SAur22_S22_L001_R2_001.fastq.gz	**PASS**	**PASS**	**FAIL**	**FAIL**	**PASS**
M16SAur22_S22_L001_R1_001.fastq.gz	**PASS**	**PASS**	**FAIL**	**FAIL**	**PASS**
M16SAur23_S23_L001_R2_001.fastq.gz	**PASS**	**PASS**	**FAIL**	**FAIL**	**PASS**
M16SAur23_S23_L001_R1_001.fastq.gz	**PASS**	**PASS**	**FAIL**	**FAIL**	**PASS**
M16SAur24_S24_L001_R2_001.fastq.gz	**PASS**	**PASS**	**FAIL**	**WARNING**	**PASS**
M16SAur24_S24_L001_R1_001.fastq.gz	**PASS**	**PASS**	**FAIL**	**WARNING**	**PASS**
M16SAur5_S5_L001_R2_001.fastq.gz	**PASS**	**PASS**	**FAIL**	**FAIL**	**PASS**
M16SAur4_S4_L001_R2_001.fastq.gz	**PASS**	**PASS**	**FAIL**	**WARNING**	**PASS**
M16SAur4_S4_L001_R1_001.fastq.gz	**PASS**	**PASS**	**FAIL**	**WARNING**	**PASS**
M16SAur5_S5_L001_R1_001.fastq.gz	**PASS**	**PASS**	**FAIL**	**WARNING**	**PASS**
M16SAur7_S7_L001_R2_001.fastq.gz	**PASS**	**PASS**	**FAIL**	**FAIL**	**PASS**
M16SAur6_S6_L001_R2_001.fastq.gz	**PASS**	**PASS**	**FAIL**	**FAIL**	**PASS**
M16SAur6_S6_L001_R1_001.fastq.gz	**PASS**	**PASS**	**FAIL**	**WARNING**	**PASS**
M16SAur7_S7_L001_R1_001.fastq.gz	**PASS**	**PASS**	**FAIL**	**FAIL**	**PASS**
M16SAur8_S8_L001_R2_001.fastq.gz	**PASS**	**PASS**	**FAIL**	**FAIL**	**PASS**
M16SAur8_S8_L001_R1_001.fastq.gz	**PASS**	**PASS**	**FAIL**	**WARNING**	**PASS**
M16SAur9_S9_L001_R2_001.fastq.gz	**FAIL**	**PASS**	**FAIL**	**FAIL**	**PASS**
M16SAurC1_S25_L001_R2_001.fastq.gz	**PASS**	**PASS**	**FAIL**	**FAIL**	**PASS**
M16SAur9_S9_L001_R1_001.fastq.gz	**PASS**	**PASS**	**FAIL**	**FAIL**	**PASS**
M16SAurC1_S25_L001_R1_001.fastq.gz	**PASS**	**PASS**	**FAIL**	**FAIL**	**PASS**
M16SAurC10_S33_L001_R2_001.fastq.gz	**PASS**	**PASS**	**FAIL**	**FAIL**	**PASS**
M16SAurC10_S33_L001_R1_001.fastq.gz	**PASS**	**PASS**	**FAIL**	**FAIL**	**PASS**
M16SAurC11_S34_L001_R2_001.fastq.gz	**PASS**	**PASS**	**FAIL**	**PASS**	**PASS**
M16SAurC12_S35_L001_R2_001.fastq.gz	**PASS**	**PASS**	**FAIL**	**FAIL**	**PASS**
M16SAurC11_S34_L001_R1_001.fastq.gz	**PASS**	**PASS**	**FAIL**	**WARNING**	**PASS**
M16SAurC12_S35_L001_R1_001.fastq.gz	**PASS**	**PASS**	**FAIL**	**FAIL**	**PASS**
M16SAurC13_S36_L001_R2_001.fastq.gz	**PASS**	**PASS**	**FAIL**	**FAIL**	**PASS**
M16SAurC13_S36_L001_R1_001.fastq.gz	**PASS**	**PASS**	**FAIL**	**FAIL**	**PASS**
M16SAurC14_S37_L001_R2_001.fastq.gz	**PASS**	**PASS**	**FAIL**	**FAIL**	**PASS**

M16SAurC15_S38_L001_R2_001.fastq.gz	**PASS**	**PASS**	**FAIL**	**FAIL**	**PASS**
M16SAurC14_S37_L001_R1_001.fastq.gz	**PASS**	**PASS**	**FAIL**	**WARNING**	**PASS**
M16SAurC15_S38_L001_R1_001.fastq.gz	**PASS**	**PASS**	**FAIL**	**FAIL**	**PASS**
M16SAurC17_S40_L001_R2_001.fastq.gz	**PASS**	**PASS**	**FAIL**	**FAIL**	**PASS**
M16SAurC16_S39_L001_R1_001.fastq.gz	**PASS**	**PASS**	**FAIL**	**WARNING**	**PASS**
M16SAurC16_S39_L001_R2_001.fastq.gz	**PASS**	**PASS**	**FAIL**	**FAIL**	**PASS**
M16SAurC17_S40_L001_R1_001.fastq.gz	**PASS**	**PASS**	**FAIL**	**FAIL**	**PASS**
M16SAurC18_S41_L001_R1_001.fastq.gz	**PASS**	**PASS**	**FAIL**	**WARNING**	**PASS**
M16SAurC18_S41_L001_R2_001.fastq.gz	**PASS**	**PASS**	**FAIL**	**FAIL**	**PASS**
M16SAurC19_S42_L001_R2_001.fastq.gz	**PASS**	**PASS**	**FAIL**	**FAIL**	**PASS**
M16SAurC20_S43_L001_R2_001.fastq.gz	**PASS**	**PASS**	**FAIL**	**FAIL**	**PASS**
M16SAurC19_S42_L001_R1_001.fastq.gz	**PASS**	**PASS**	**FAIL**	**FAIL**	**PASS**
M16SAurC20_S43_L001_R1_001.fastq.gz	**PASS**	**PASS**	**FAIL**	**FAIL**	**PASS**
M16SAurC21_S44_L001_R2_001.fastq.gz	**PASS**	**PASS**	**FAIL**	**FAIL**	**PASS**
M16SAurC21_S44_L001_R1_001.fastq.gz	**PASS**	**PASS**	**FAIL**	**FAIL**	**PASS**
M16SAurC22_S45_L001_R2_001.fastq.gz	**PASS**	**PASS**	**FAIL**	**FAIL**	**PASS**
M16SAurC22_S45_L001_R1_001.fastq.gz	**PASS**	**PASS**	**FAIL**	**WARNING**	**PASS**
M16SAurC23_S46_L001_R2_001.fastq.gz	**PASS**	**PASS**	**FAIL**	**FAIL**	**PASS**
M16SAurC23_S46_L001_R1_001.fastq.gz	**PASS**	**PASS**	**FAIL**	**FAIL**	**PASS**
M16SAurC24_S47_L001_R2_001.fastq.gz	**PASS**	**PASS**	**FAIL**	**FAIL**	**PASS**
M16SAurC24_S47_L001_R1_001.fastq.gz	**PASS**	**PASS**	**FAIL**	**WARNING**	**PASS**
M16SAurC4_S27_L001_R2_001.fastq.gz	**PASS**	**PASS**	**FAIL**	**WARNING**	**PASS**
M16SAurC3_S26_L001_R2_001.fastq.gz	**PASS**	**PASS**	**FAIL**	**FAIL**	**PASS**
M16SAurC4_S27_L001_R1_001.fastq.gz	**PASS**	**PASS**	**FAIL**	**WARNING**	**PASS**
M16SAurC3_S26_L001_R1_001.fastq.gz	**PASS**	**PASS**	**FAIL**	**FAIL**	**PASS**
M16SAurC5_S28_L001_R2_001.fastq.gz	**PASS**	**PASS**	**FAIL**	**FAIL**	**PASS**
M16SAurC5_S28_L001_R1_001.fastq.gz	**PASS**	**PASS**	**FAIL**	**WARNING**	**PASS**
M16SAurC7_S30_L001_R2_001.fastq.gz	**PASS**	**PASS**	**FAIL**	**WARNING**	**PASS**
M16SAurC7_S30_L001_R1_001.fastq.gz	**PASS**	**PASS**	**FAIL**	**WARNING**	**PASS**
M16SAurC8_S31_L001_R2_001.fastq.gz	**PASS**	**PASS**	**FAIL**	**FAIL**	**PASS**
M16SAurC8_S31_L001_R1_001.fastq.gz	**PASS**	**PASS**	**FAIL**	**FAIL**	**PASS**

M16SAurC9_S32_L001_R2_001.fastq.gz	**PASS**	**PASS**	**FAIL**	**FAIL**	**PASS**
M16SAurC9_S32_L001_R1_001.fastq.gz	**PASS**	**PASS**	**FAIL**	**FAIL**	**PASS**

Name	Sequence Length Distribution	Adapter Content	Overrepresented Sequences	Sequence Duplication Levels
M16SAur1_S1_L001_R1_001.fastq.gz	**WARNING**	**PASS**	**FAIL**	**FAIL**
M16SAur1_S1_L001_R2_001.fastq.gz	**WARNING**	**PASS**	**FAIL**	**FAIL**
M16SAur10_S10_L001_R2_001.fastq.gz	**WARNING**	**PASS**	**FAIL**	**FAIL**
M16SAur11_S11_L001_R2_001.fastq.gz	**WARNING**	**PASS**	**FAIL**	**FAIL**
M16SAur11_S11_L001_R1_001.fastq.gz	**WARNING**	**PASS**	**FAIL**	**FAIL**
M16SAur10_S10_L001_R1_001.fastq.gz	**WARNING**	**PASS**	**FAIL**	**FAIL**
M16SAur12_S12_L001_R2_001.fastq.gz	**WARNING**	**PASS**	**FAIL**	**FAIL**
M16SAur12_S12_L001_R1_001.fastq.gz	**WARNING**	**PASS**	**FAIL**	**FAIL**
M16SAur13_S13_L001_R2_001.fastq.gz	**WARNING**	**PASS**	**FAIL**	**FAIL**
M16SAur13_S13_L001_R1_001.fastq.gz	**WARNING**	**PASS**	**FAIL**	**FAIL**
M16SAur14_S14_L001_R2_001.fastq.gz	**WARNING**	**PASS**	**FAIL**	**FAIL**
M16SAur15_S15_L001_R2_001.fastq.gz	**WARNING**	**PASS**	**FAIL**	**FAIL**
M16SAur14_S14_L001_R1_001.fastq.gz	**WARNING**	**PASS**	**FAIL**	**FAIL**
M16SAur15_S15_L001_R1_001.fastq.gz	**WARNING**	**PASS**	**FAIL**	**FAIL**
M16SAur16_S16_L001_R2_001.fastq.gz	**WARNING**	**PASS**	**FAIL**	**FAIL**
M16SAur16_S16_L001_R1_001.fastq.gz	**WARNING**	**PASS**	**FAIL**	**FAIL**
M16SAur17_S17_L001_R2_001.fastq.gz	**WARNING**	**PASS**	**FAIL**	**FAIL**
M16SAur17_S17_L001_R1_001.fastq.gz	**WARNING**	**PASS**	**FAIL**	**FAIL**
M16SAur18_S18_L001_R2_001.fastq.gz	**WARNING**	**PASS**	**FAIL**	**FAIL**
M16SAur18_S18_L001_R1_001.fastq.gz	**WARNING**	**PASS**	**FAIL**	**FAIL**
M16SAur19_S19_L001_R2_001.fastq.gz	**WARNING**	**PASS**	**FAIL**	**FAIL**
M16SAur19_S19_L001_R1_001.fastq.gz	**WARNING**	**PASS**	**FAIL**	**FAIL**
M16SAur2_S2_L001_R2_001.fastq.gz	**WARNING**	**PASS**	**FAIL**	**FAIL**
M16SAur2_S2_L001_R1_001.fastq.gz	**WARNING**	**PASS**	**FAIL**	**FAIL**
M16SAur20_S20_L001_R2_001.fastq.gz	**WARNING**	**PASS**	**FAIL**	**FAIL**
M16SAur21_S21_L001_R2_001.fastq.gz	**WARNING**	**PASS**	**FAIL**	**FAIL**
M16SAur21_S21_L001_R1_001.fastq.gz	**WARNING**	**PASS**	**FAIL**	**FAIL**

M16SAur20_S20_L001_R1_001.fastq.gz	**WARNING**	**PASS**	**FAIL**	**FAIL**
M16SAur22_S22_L001_R2_001.fastq.gz	**WARNING**	**PASS**	**FAIL**	**FAIL**
M16SAur22_S22_L001_R1_001.fastq.gz	**WARNING**	**PASS**	**FAIL**	**FAIL**
M16SAur23_S23_L001_R2_001.fastq.gz	**WARNING**	**PASS**	**FAIL**	**FAIL**
M16SAur23_S23_L001_R1_001.fastq.gz	**WARNING**	**PASS**	**FAIL**	**FAIL**
M16SAur24_S24_L001_R2_001.fastq.gz	**WARNING**	**PASS**	**FAIL**	**FAIL**
M16SAur24_S24_L001_R1_001.fastq.gz	**WARNING**	**PASS**	**FAIL**	**FAIL**
M16SAur5_S5_L001_R2_001.fastq.gz	**WARNING**	**PASS**	**FAIL**	**FAIL**
M16SAur4_S4_L001_R2_001.fastq.gz	**WARNING**	**PASS**	**FAIL**	**FAIL**
M16SAur4_S4_L001_R1_001.fastq.gz	**WARNING**	**PASS**	**FAIL**	**FAIL**
M16SAur5_S5_L001_R1_001.fastq.gz	**WARNING**	**PASS**	**FAIL**	**FAIL**
M16SAur7_S7_L001_R2_001.fastq.gz	**WARNING**	**PASS**	**FAIL**	**FAIL**
M16SAur6_S6_L001_R2_001.fastq.gz	**WARNING**	**PASS**	**FAIL**	**FAIL**
M16SAur6_S6_L001_R1_001.fastq.gz	**WARNING**	**PASS**	**FAIL**	**FAIL**
M16SAur7_S7_L001_R1_001.fastq.gz	**WARNING**	**PASS**	**FAIL**	**FAIL**
M16SAur8_S8_L001_R2_001.fastq.gz	**WARNING**	**PASS**	**FAIL**	**FAIL**
M16SAur8_S8_L001_R1_001.fastq.gz	**WARNING**	**PASS**	**FAIL**	**FAIL**
M16SAur9_S9_L001_R2_001.fastq.gz	**WARNING**	**PASS**	**FAIL**	**FAIL**
M16SAurC1_S25_L001_R2_001.fastq.gz	**WARNING**	**PASS**	**FAIL**	**FAIL**
M16SAur9_S9_L001_R1_001.fastq.gz	**WARNING**	**PASS**	**FAIL**	**FAIL**
M16SAurC1_S25_L001_R1_001.fastq.gz	**WARNING**	**PASS**	**FAIL**	**FAIL**
M16SAurC10_S33_L001_R2_001.fastq.gz	**WARNING**	**PASS**	**FAIL**	**FAIL**
M16SAurC10_S33_L001_R1_001.fastq.gz	**WARNING**	**PASS**	**FAIL**	**FAIL**
M16SAurC11_S34_L001_R2_001.fastq.gz	**WARNING**	**PASS**	**FAIL**	**FAIL**
M16SAurC12_S35_L001_R2_001.fastq.gz	**WARNING**	**PASS**	**FAIL**	**FAIL**
M16SAurC11_S34_L001_R1_001.fastq.gz	**WARNING**	**PASS**	**FAIL**	**FAIL**
M16SAurC12_S35_L001_R1_001.fastq.gz	**WARNING**	**PASS**	**FAIL**	**FAIL**
M16SAurC13_S36_L001_R2_001.fastq.gz	**WARNING**	**PASS**	**FAIL**	**FAIL**
M16SAurC13_S36_L001_R1_001.fastq.gz	**WARNING**	**PASS**	**FAIL**	**FAIL**
M16SAurC14_S37_L001_R2_001.fastq.gz	**WARNING**	**PASS**	**FAIL**	**FAIL**
M16SAurC15_S38_L001_R2_001.fastq.gz	**WARNING**	**PASS**	**FAIL**	**FAIL**

M16SAurC14_S37_L001_R1_001.fastq.gz	**WARNING**	**PASS**	**FAIL**	**FAIL**
M16SAurC15_S38_L001_R1_001.fastq.gz	**WARNING**	**PASS**	**FAIL**	**FAIL**
M16SAurC17_S40_L001_R2_001.fastq.gz	**WARNING**	**PASS**	**FAIL**	**FAIL**
M16SAurC16_S39_L001_R1_001.fastq.gz	**WARNING**	**PASS**	**FAIL**	**FAIL**
M16SAurC16_S39_L001_R2_001.fastq.gz	**WARNING**	**PASS**	**FAIL**	**FAIL**
M16SAurC17_S40_L001_R1_001.fastq.gz	**WARNING**	**PASS**	**FAIL**	**FAIL**
M16SAurC18_S41_L001_R1_001.fastq.gz	**WARNING**	**PASS**	**FAIL**	**FAIL**
M16SAurC18_S41_L001_R2_001.fastq.gz	**WARNING**	**PASS**	**FAIL**	**FAIL**
M16SAurC19_S42_L001_R2_001.fastq.gz	**WARNING**	**PASS**	**FAIL**	**FAIL**
M16SAurC20_S43_L001_R2_001.fastq.gz	**WARNING**	**PASS**	**FAIL**	**FAIL**
M16SAurC19_S42_L001_R1_001.fastq.gz	**WARNING**	**PASS**	**FAIL**	**FAIL**
M16SAurC20_S43_L001_R1_001.fastq.gz	**WARNING**	**PASS**	**FAIL**	**FAIL**
M16SAurC21_S44_L001_R2_001.fastq.gz	**WARNING**	**PASS**	**FAIL**	**FAIL**
M16SAurC21_S44_L001_R1_001.fastq.gz	**WARNING**	**PASS**	**FAIL**	**FAIL**
M16SAurC22_S45_L001_R2_001.fastq.gz	**WARNING**	**PASS**	**FAIL**	**FAIL**
M16SAurC22_S45_L001_R1_001.fastq.gz	**WARNING**	**PASS**	**FAIL**	**FAIL**
M16SAurC23_S46_L001_R2_001.fastq.gz	**WARNING**	**PASS**	**FAIL**	**FAIL**
M16SAurC23_S46_L001_R1_001.fastq.gz	**WARNING**	**PASS**	**FAIL**	**FAIL**
M16SAurC24_S47_L001_R2_001.fastq.gz	**WARNING**	**PASS**	**FAIL**	**FAIL**
M16SAurC24_S47_L001_R1_001.fastq.gz	**WARNING**	**PASS**	**FAIL**	**FAIL**
M16SAurC4_S27_L001_R2_001.fastq.gz	**WARNING**	**PASS**	**FAIL**	**FAIL**
M16SAurC3_S26_L001_R2_001.fastq.gz	**WARNING**	**PASS**	**FAIL**	**FAIL**
M16SAurC4_S27_L001_R1_001.fastq.gz	**WARNING**	**PASS**	**FAIL**	**FAIL**
M16SAurC3_S26_L001_R1_001.fastq.gz	**WARNING**	**PASS**	**FAIL**	**FAIL**
M16SAurC5_S28_L001_R2_001.fastq.gz	**WARNING**	**PASS**	**FAIL**	**FAIL**
M16SAurC5_S28_L001_R1_001.fastq.gz	**WARNING**	**PASS**	**FAIL**	**FAIL**
M16SAurC7_S30_L001_R2_001.fastq.gz	**WARNING**	**PASS**	**FAIL**	**FAIL**
M16SAurC7_S30_L001_R1_001.fastq.gz	**WARNING**	**PASS**	**FAIL**	**FAIL**
M16SAurC8_S31_L001_R2_001.fastq.gz	**WARNING**	**PASS**	**FAIL**	**FAIL**
M16SAurC8_S31_L001_R1_001.fastq.gz	**WARNING**	**PASS**	**FAIL**	**FAIL**
M16SAurC9_S32_L001_R2_001.fastq.gz	**WARNING**	**PASS**	**FAIL**	**FAIL**

M16SAurC9_S32_L001_R1_001.fastq.gz	**WARNING**	**PASS**	**FAIL**	**FAIL**

The FASTQ quality check task is performed by nine analysis modules. The table above provides a quick evaluation of whether the results of each module seem entirely normal (pass), sightly abnormal (warning) or very unusual (fail). Note that these evaluations must be taken in the context of what is expected from the library. For example, some experiments may be expected to produce libraries which are biased in particular ways. Therefore, the summary evaluations should be treated as pointers that guide the preprocessing of the libraries.

Analysis Parameters

Parameter	Value
Chart Read Length Binning	true
Provide Adapter Sequences	false
Provide Contaminant Sequences	false

References

- FastQC: A Quality Control tool for High Thoughput Sequence Data. Andrews S. 2018. www.bioinformatics.babraham.ac.uk/projects/fastqc.
- OmicsBox - Bioinformatics made easy. BioBam Bioinformatics (Version 2.0.36). March 3, 2019. www.biobam.com/omicsbox.
