# Supplementary material for: Impact of Mango Bagasse and Peel Confectionery Rich in Dietary Fiber on Gut Microbiota, Metabolite Profiles, and Genetic Regulation in High-Fat-Diet-Fed Wistar Rats
Source: Nutrients. 2025 Dec 2;17(23):3780. doi: 10.3390/nu17233780 (PMC12694227; doi:10.3390/nu17233780)
Supplement: Supplementary file 1 [file nutrients-17-03780-s001.zip › Supplementary material.pdf]

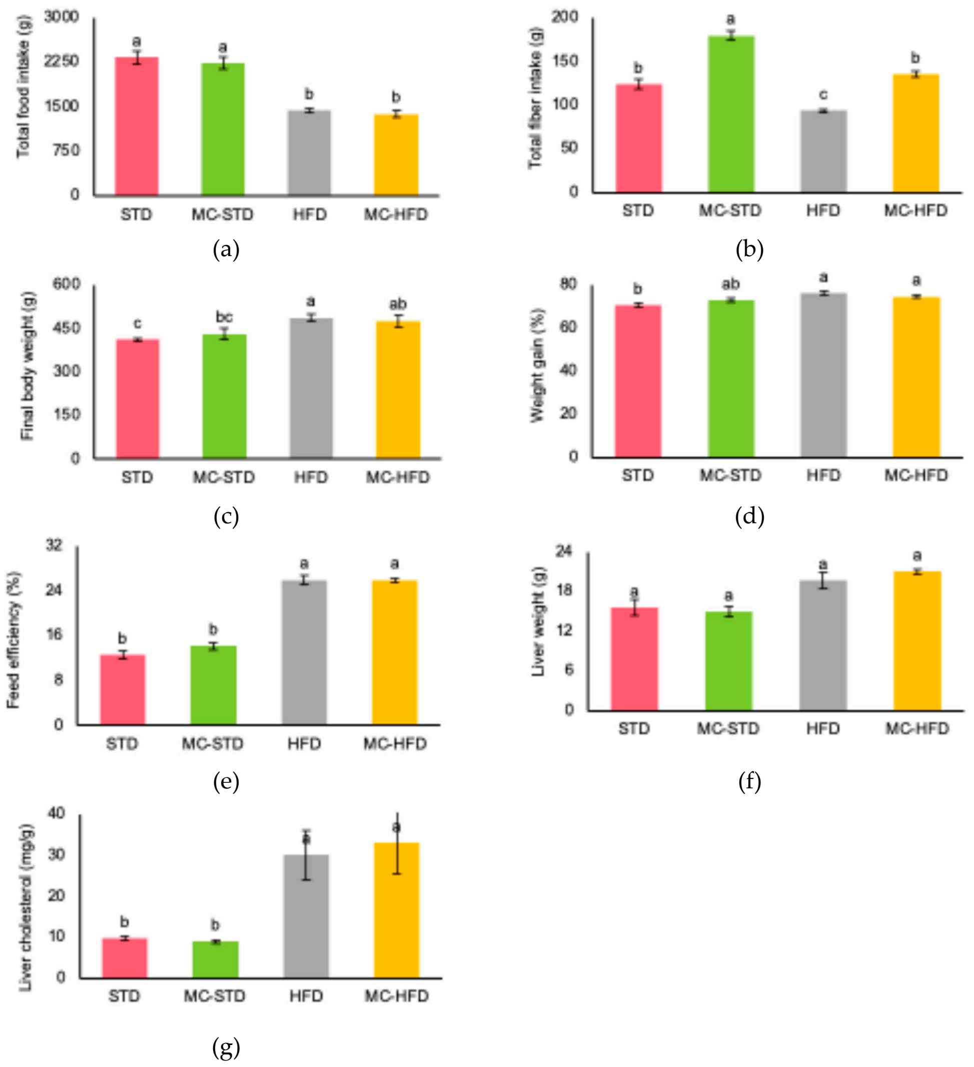

Figure S1. Eating behavior and body composition. (**a**) Total food intake; (**b**) total fiber intake; (**c**) final body weight (week 11); (**d**) weight gain; (**e**) feed efficiency; (**f**) liver weight; and (**g**) liver cholesterol.

Table S1. Principal component analysis (PCA) cumulative loadings for variables associated with dietary intake and metabolic parameters.

Variable	Cumulative loading on PC1 and PC2
Total fiber intake	0.9873
Feed efficiency	0.9551
Total MC intake	0.9531
Diet type	0.9477
Total food intake	0.8435
Liver cholesterol	0.7224
Body weight	0.6462

Cumulative loadings of the variables contributing to the first two principal components (PC1 and PC2) obtained from the PCA integrating dietary intake and metabolic parameters.

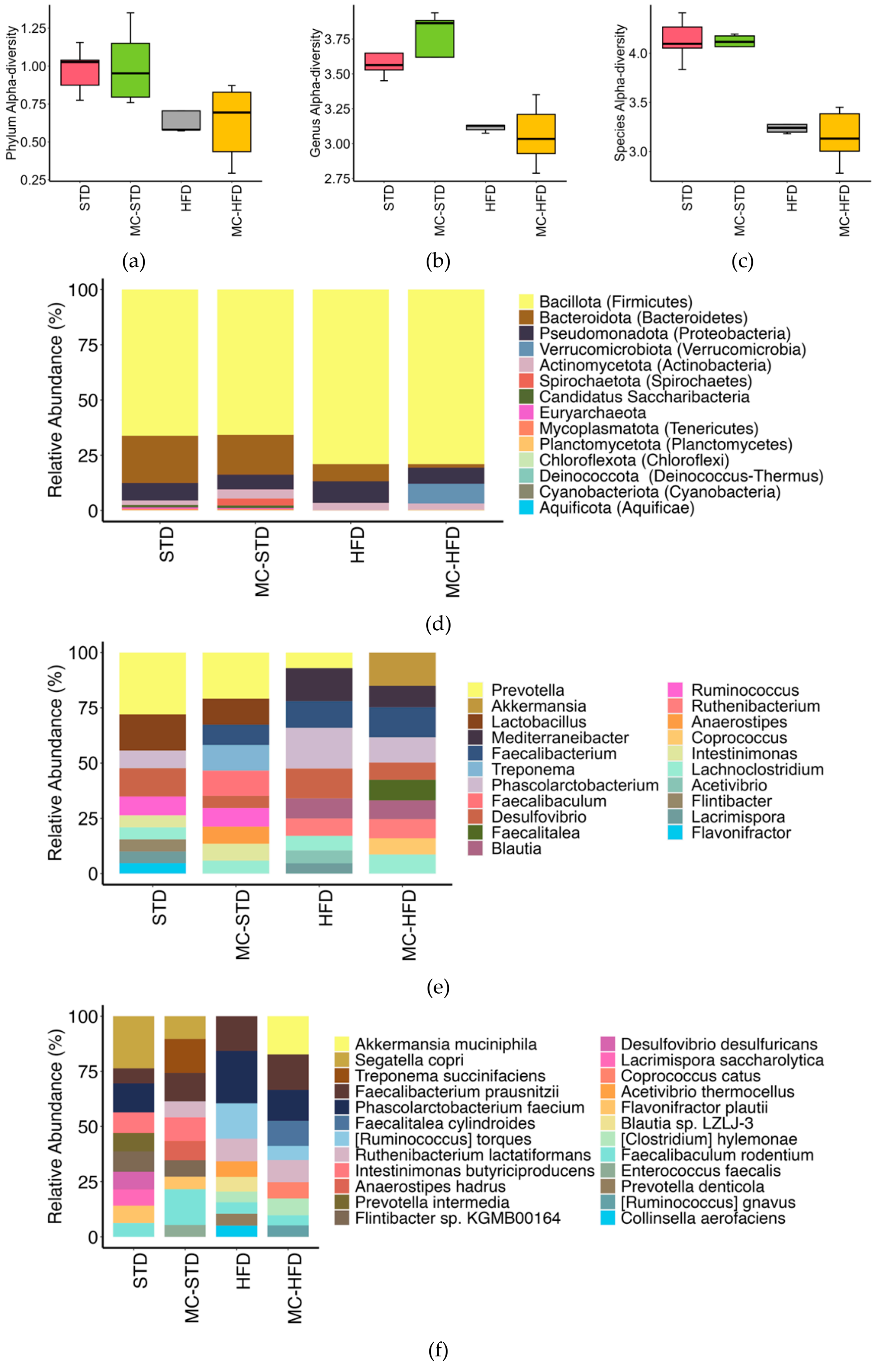

Figure S2. Alpha diversity and relative abundances of gut microbiota across taxonomic levels. (**a**) Phylum alpha diversity; (**b**) genus alpha diversity; (**c**) species alpha diversity; (**d**) phylum relative abundances; (**e**) genus relative abundances; and (**f**) species relative abundances.

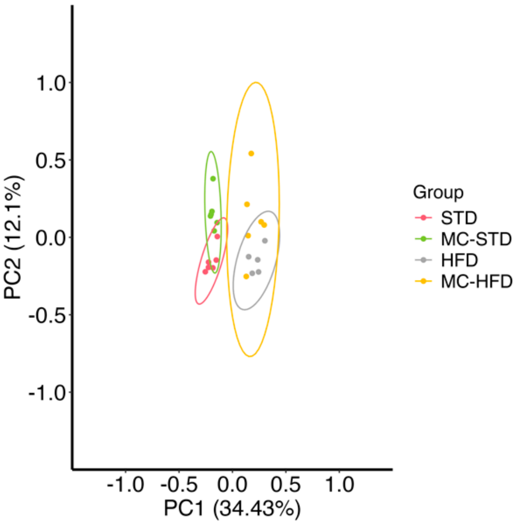

(a)

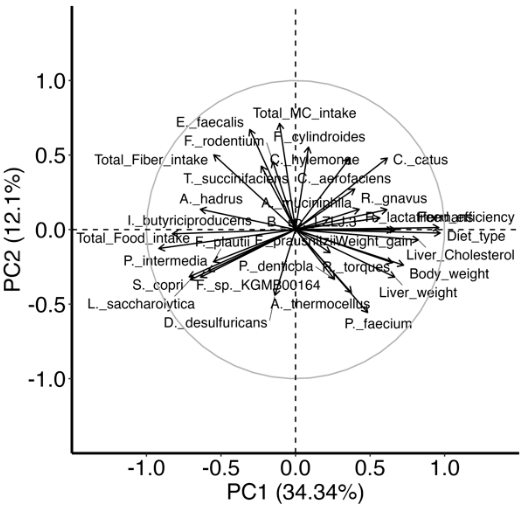

(b)

Figure S3. Principal component analysis (PCA) integrating gut microbiota, fiber intake, and body composition variables. (**a**) PCA score plot (PC1 vs. PC2); and (**b**) PCA scatter plot showing variable contribution.

Table S2. Principal component analysis (PCA) cumulative loadings for variables associated with gut microbiota composition, dietary intake, and metabolic parameters.

Variable	Cumulative loading on PC1 and PC2
Diet type	0.9422
Feed efficiency	0.9236
Total food intake	0.8568
Liver cholesterol	0.6842
I. butyriciproducens	0.6838
L. saccharolytica	0.6129
S. copri	0.6097
C. catus	0.6095
Body weight	0.5826
Total fiber intake	0.5524
Liver weight	0.5454
E. faecalis	0.5446
P. faecium	0.5439
Total MC intake	0.5182
F._sp._KGMB00164	0.5086

Cumulative loadings of the variables contributing to the first two principal components (PC1 and PC2) obtained from the PCA integrating gut microbiota, dietary intake, and metabolic parameters.

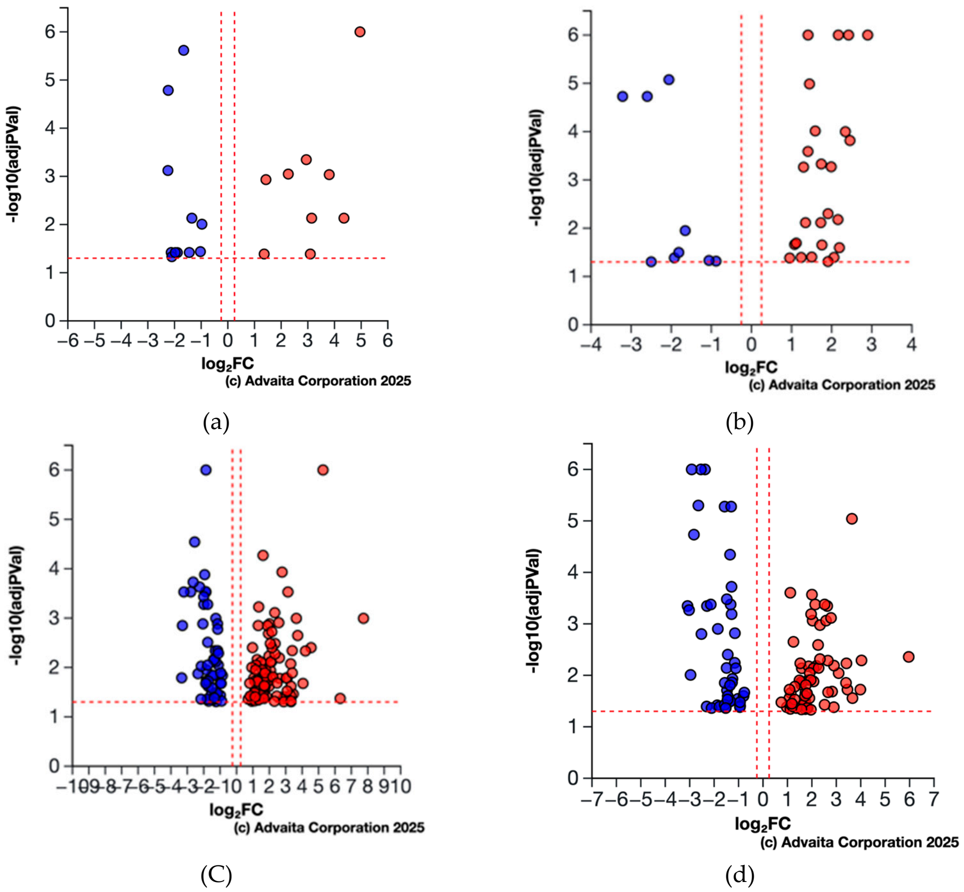

Figure S4. Volcano plots of differentially expressed genes across contrasts. (**a**) HFD vs. STD; (**b**) MC-STD vs. HFD; (**c**) MC-HFD vs. STD; and (**d**) MC-HFD vs. MC-STD.

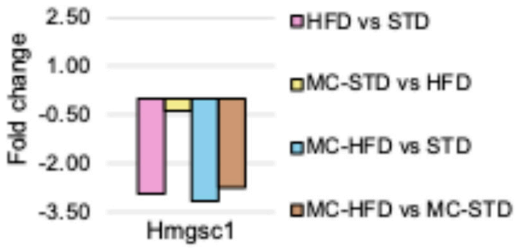

(a)

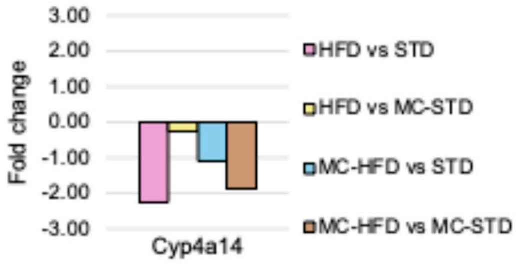

(b)

Figure S5. Differential gene expression across contrasts validated by qPCR. (**a**) Hmgcs1; and (**b**) Cyp4a14.
